# When the heart cannot keep pace: case report of acute myocarditis in a recreational marathon runner

**DOI:** 10.1093/ehjcr/ytag245

**Published:** 2026-03-26

**Authors:** Lisa Marie Dertinger, Frank Ruschitzka, Jan Alphard Kleeberger

**Affiliations:** Department of Cardiology, University Heart Center, University Hospital Zurich, University of Zurich, Raemistrasse 100, 8091 Zurich, Switzerland; Department of Cardiology, University Heart Center, University Hospital Zurich, University of Zurich, Raemistrasse 100, 8091 Zurich, Switzerland; Center for Translational and Experimental Cardiology (CTEC), Department of Cardiology, University Hospital Zurich and University of Zurich, Wagistrasse 12, 8952 Schlieren, Switzerland; Department of Cardiology, University Heart Center, University Hospital Zurich, University of Zurich, Raemistrasse 100, 8091 Zurich, Switzerland; Center for Translational and Experimental Cardiology (CTEC), Department of Cardiology, University Hospital Zurich and University of Zurich, Wagistrasse 12, 8952 Schlieren, Switzerland

**Keywords:** Myocarditis, Sports medicine, Biomarkers, Cardiac magnetic resonance imaging, Sports-related cardiac event, Case report

## Abstract

**Background:**

Marathon running is associated with transient elevations in cardiac and skeletal muscle biomarkers, which generally normalize within 72 h. However, persistent or extreme biomarker elevations may indicate underlying pathology, such as myocarditis. Early recognition and guideline-based management are essential to prevent complications and guide safe return to exercise.

**Case summary:**

A 21-year-old recreational marathon runner collapsed towards the end of a marathon race. On presentation, he exhibited markedly elevated cardiac and muscle biomarkers, including high-sensitivity cardiac troponin T, creatine kinase, creatine kinase–myocardial band, and myoglobin. The initial electrocardiogram (ECG) demonstrated a tachycardic sinus rhythm with T-wave inversion in Lead III, without evidence of significant depolarization or repolarization abnormalities. Cardiac Magnetic Resonance Imaging (MRI) confirmed acute myocarditis. The patient was managed supportively with intravenous fluids and non-steroidal anti-inflammatory drug analgesics, remained haemodynamically stable, and experienced no arrhythmias or chest pain. At 3-month follow-up, he reported good functional capacity, and both echocardiography and cardiac MRI were completely normal, demonstrating full resolution of myocardial inflammation.

**Discussion:**

This case highlights the diagnostic challenge of interpreting elevated cardiac biomarkers in endurance athletes. Observational studies indicate that biomarker elevations often correlate with exercise duration rather than with demographic or training-related factors. Cardiac MRI remains essential for non-invasive diagnosis, risk stratification, and follow-up. Importantly, adherence to guideline-based management, i.e. temporary abstinence from competitive sports, monitoring of biomarkers, and serial imaging, can lead to excellent outcomes, as exemplified by the complete normalization observed in this patient. Non-steroidal anti-inflammatory drugs should be used with caution in amateur endurance athletes due to their potential to aggravate myocarditis.

Learning points

- Transient elevations in troponin, creatine kinase, and myoglobin are common after marathon running, but persistent or marked elevations with imaging abnormalities indicate pathology.
- Pre-race non-steroidal anti-inflammatory drug use may worsen subclinical myocarditis in athletes, highlighting the need for caution with these drugs, particularly in the context of recent infection or prodromal symptoms.
- Cardiac MRI is essential for diagnosis, risk stratification, and guiding return to exercise.

## Introduction

Endurance exercise, such as marathon running, has well-established cardiovascular benefits but can also pose unique diagnostic challenges when athletes present with symptoms suggestive of cardiac pathology. Transient elevations in cardiac biomarkers, particularly high-sensitivity cardiac troponin T (hs-cTnT) and creatine kinase (CK), are frequently observed after prolonged exertion and are typically benign, resolving within 72 h. However, distinguishing physiological adaptation from underlying myocardial injury remains critical, especially in the context of persistent biomarker elevation or accompanying symptoms.

Myocarditis is a potentially life-threatening condition and a recognized cause of sudden cardiac death in young athletes. It may present subtly, often mimicking benign post-exercise syndromes or viral illness. Cardiac magnetic resonance imaging (CMR) has emerged as the gold standard for non-invasive diagnosis, enabling the detection of myocardial inflammation, oedema, and fibrosis based on Lake Louise criteria.

Here, we present a case of a young, previously healthy recreational marathon runner who collapsed during a race and was ultimately diagnosed with acute myocarditis. This case underscores the importance of clinical vigilance in athletic populations, the role of CMR in diagnostic clarification, and the relevance of guideline-based recommendations for return-to-sport decisions.

## Summary figure

**Figure:**
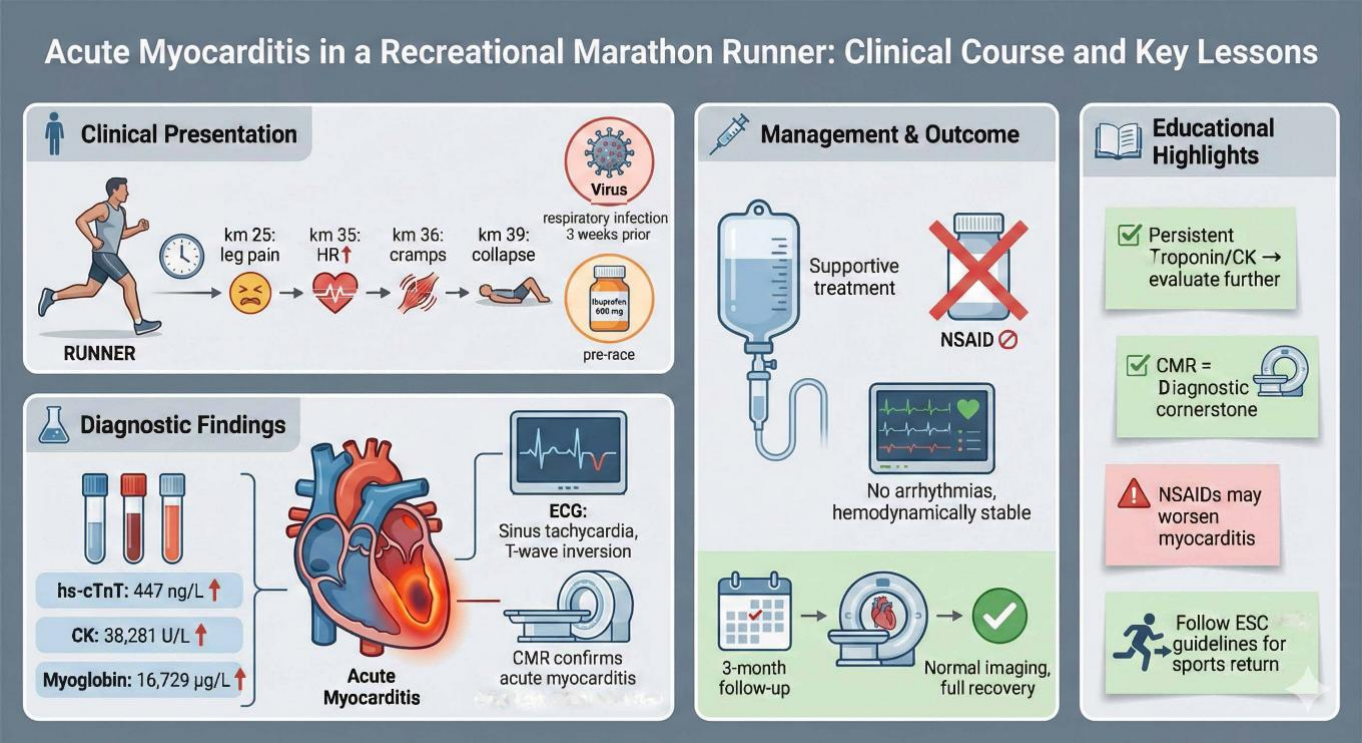


## Case presentation

### Pre-race history

The patient is a 21-year-old male university student and an experienced recreational runner, who trained 4–6 h per week (including 1–2× tennis, 1–2× gym sessions with cycling, 1–2× long-distance runs, and occasional football). He had completed three half-marathons previously and has engaged in endurance sports since childhood without prior complications during longer runs. Three weeks prior to the marathon, he experienced a respiratory infection but continued his exercise regimen. His past medical history includes attention deficit disorder, treated intermittently with methylphenidate 10 mg only during exam periods, and regular Ginkgo supplementation. He occasionally uses snus on weekends, consumes alcohol sparingly, and denies drug use. The family history is negative for cardiovascular events.

### Collapse and emergency department evaluation

On marathon day, the patient felt well prepared and symptom-free, after having taken a single 600 mg dose of ibuprofen the previous day for a twisted foot. Around Kilometre 25, he experienced stabbing foot pain and leg heaviness. By Kilometre 35, he noticed a marked increase in heart rate. After attempting to pause at Kilometre 36, he developed full-body cramps but tried to continue running. His recollection from around Kilometre 25 is very vague, and he has no memory after Kilometre 36, regaining consciousness only in the ambulance. According to external observations, the patient was last seen running at approximately Kilometre 39. Shortly afterwards, he presented at a medical station in a state of severe confusion and with intense back pain, without evidence of a fall.

Emergency medical services documented an initial inter-arm blood pressure difference of 40 mmHg. Analgesics (fentanyl 50 µg) did not relieve his back pain, and he was admitted via the resuscitation room with suspected aortic dissection.

**Figure 1:**
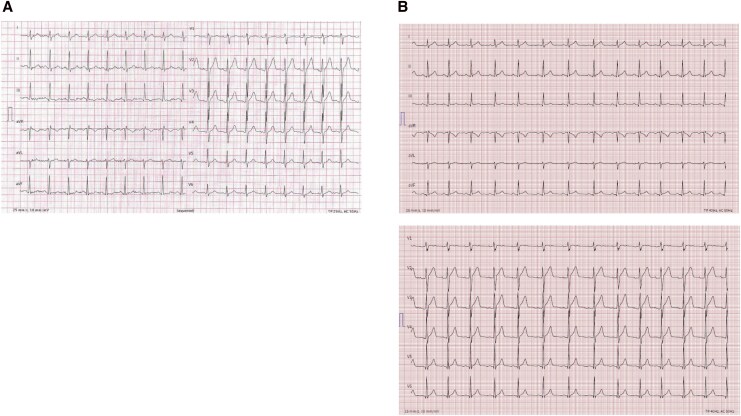
ECGs of the patient. (*A*) Day 1, at presentation. (*B*) Day 1, 5 h after presentation.

**Figure 2:**
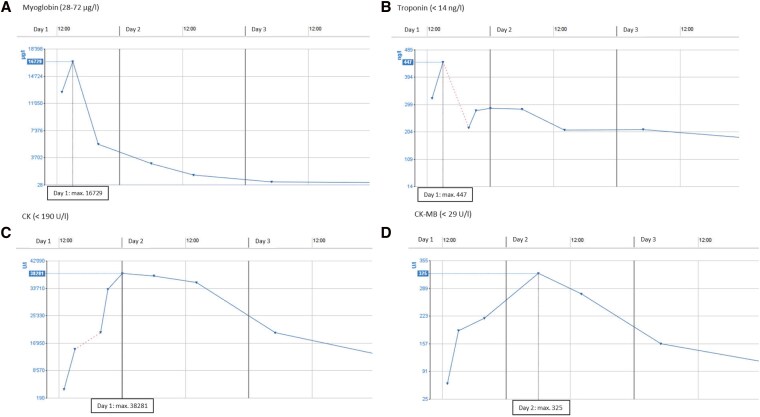
Laboratory values during the hospitalization. (*A*) Myoglobin, (*B*) troponin T, (*C*) creatine kinase, and (*D*) creatine kinase–muscle–brain subunits.

The initial electrocardiogram (ECG) was affected by artefacts but demonstrated a tachycardic sinus rhythm with T-wave inversion in Lead III, without evidence of significant depolarization or repolarization abnormalities (*Figure 1A*). A serial ECG showed normal sinus rhythm with normalization of the T-wave in Lead III (*Figure 1B*). Computed tomography (CT) angiography excluded aortic dissection, pulmonary embolism, and coronary artery disease. Laboratory testing revealed markedly elevated cardiac biomarkers (*Table 1*; *Figure 2*). N-terminal pro-B-type natriuretic peptide (NT-proBNP), electrolytes, and C-reactive protein were within normal limits. On admission, serum creatinine was elevated at 1.55 mg/dL but returned to normal within the same day and remained stable thereafter. A spot urine examination revealed no casts suggestive of acute tubular necrosis in the context of presumed rhabdomyolysis.

**Table 1:** Peak laboratory values

Peak laboratory values		
Myoglobin	16729 U/L	At 2 h
hs-cTnT	447 ng/L	At 2 h
CK	38281 U/L	At 11 h
CK–MB	325 U/L	At 17 h
LDH	1079 U/L	At 25 h

### In-hospital management

**Figure 3:**
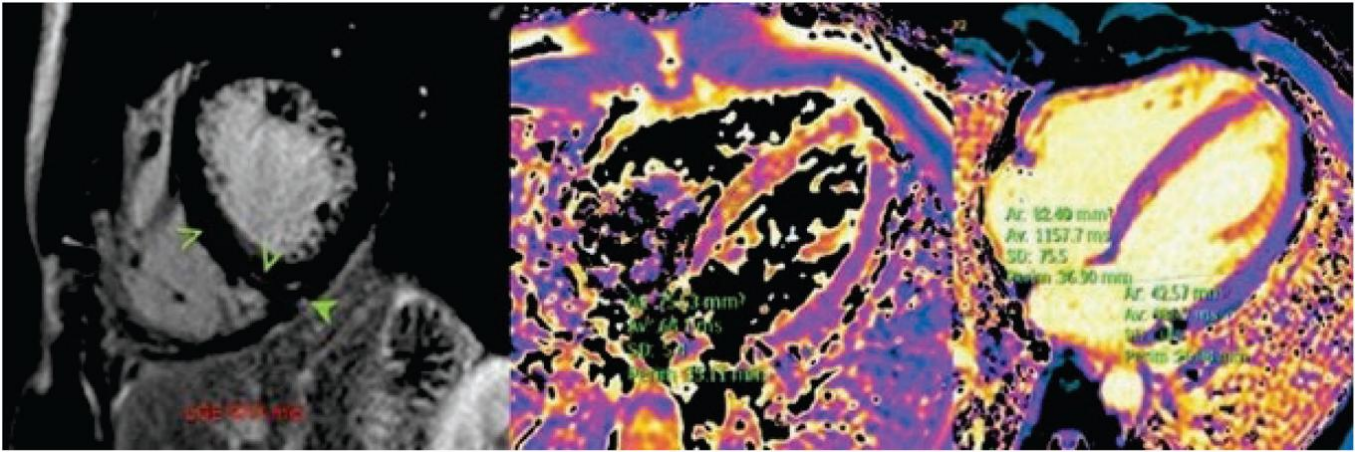
Representative images of the cardiac MRI of the patient. Delayed gadolinium enhancement sequences shown in the figure.

Due to suspected myocardial injury in the context of markedly elevated cardiac biomarkers during rhabdomyolysis and a possibly dilated right ventricle on echocardiography, the patient underwent further evaluation with cardiac Magnetic Resonance Imaging (MRI), which confirmed acute myocarditis (*Figure 3*). Treatment was primarily supportive, consisting of intravenous fluids and non-steroidal anti-inflammatory drug (NSAID) analgesics. Continuous monitoring revealed no arrhythmias, and the patient remained free of chest pain. Extended workup, including viral serologies [hepatitis A, B, and C, Human Immunodefiency Virus (HIV), cytomegalovirus (CMV), epstein-barr virus (EBV), Enterovirus] and rheumatologic screening [complement C3/C4, anti nuclear antibodies (ANA), anti-Neutrophil cytoplasmatic antibodies (ANCA), interleukin-6 (IL-6), interleukin-1β (IL-1β), tumor necrosis factor (TNF)-α], was unremarkable.

### Discharge and outpatient follow-up

The patient was discharged after 3 days with a recommendation for complete abstinence from sports for 3 months. At 3-month follow-up, he reported overall well-being and good functional capacity in daily activities. He occasionally experienced exertional dyspnoea and palpitations, without ever experiencing chest pain.

Laboratory tests were within normal limits. Echocardiography demonstrated a normal-sized left ventricle with preserved ejection fraction (LVEF; 58%) and normal longitudinal deformation. A slight increase in a small pericardial effusion in the atrioventricular (AV) groove was noted on the parasternal short-axis (PSAX) view, without haemodynamic significance (likely related to imaging plane). Follow-up cardiac MRI showed complete normalization of mapping times, with no evidence of myocardial oedema, no detectable late gadolinium enhancement (LGE), and no significant pericardial effusion. Ambulatory Holter monitoring showed no significant arrhythmias for 4 days.

## Discussion

### Case implications

This case highlights the diagnostic challenge of interpreting elevated cardiac and skeletal muscle biomarkers in a young marathon runner presenting with collapse, rhabdomyolysis, and imaging-confirmed acute myocarditis.

The 2025 European Society of Cardiology (ESC) guidelines recommend diagnosing myocarditis based on a compatible clinical presentation plus additional diagnostic criteria, including newly abnormal ECG, persistent troponin elevation, unexplained ventricular dysfunction, or CMR findings of myocardial inflammation. A definite diagnosis requires more than one additional criterion, although a single criterion may justify clinical suspicion if alternative causes are excluded.^1,2^

In this patient, persistent troponin elevation in combination with typical CMR features met the diagnostic criteria for myocarditis.

### Myocardial injury

Myocardial injury can result from ischaemic or non-ischaemic mechanisms. Ischaemic causes include coronary thrombosis, impaired myocardial perfusion, or increased myocardial oxygen demand, while non-ischaemic causes comprise cardiac and systemic conditions as well as physiological stressors, such as prolonged or intensive exercise.^3^ Myocardial injury is defined by cardiac troponin (cTn) concentrations exceeding the 99th percentile upper reference limit (URL).^3–7^ It may be classified as acute, indicated by a dynamic rise and/or fall of cTn values, or chronic, in the presence of persistently elevated troponin levels.

### Biomarker dynamics in athletes

Cardiac troponins I (cTnI) and T (cTnT) are components of the contractile apparatus of myocardial cells and are expressed almost exclusively in the heart.^3–5^ Elevations of cTnI, which was not measured in this case initially, generally do not occur following injury to non-cardiac tissues. However, transient increases have been observed after ultra-endurance exercise, particularly in athletes with exercise-induced dehydration, likely reflecting release of troponin from the myocardial cytosolic pool rather than true myocyte necrosis, possibly mediated by increased cell membrane permeability.^8^ In contrast, cTnT may occasionally rise in skeletal muscle injury, potentially complicating interpretation in athletes with marked muscular stress.^9–13^

Although elevations in cTn indicate cardiomyocyte injury, they do not reveal the underlying pathophysiological process. In athletes, transient increases may occur due to physiological stress, mechanical stretch, or cytosolic release without true myocyte necrosis.^3,14–16^

Several mechanisms have been proposed for troponin release from cardiomyocytes, including normal myocardial cell turnover, apoptosis, release of cTn degradation products, increased membrane permeability, formation and shedding of membranous blebs, and myocyte necrosis.^13,17^ Clinically, it is currently not possible to determine which mechanism accounts for a given cTn elevation.^18^

Both cTnI and cTnT, particularly when measured using high-sensitivity assays, are the preferred biomarkers for evaluating myocardial injury, while other markers, such as creatine kinase–myocardial band (CK–MB), are less sensitive and specific.^3–5,7,19^

These considerations are particularly relevant in endurance athletes, among whom transient elevations of cardiac biomarkers are common after prolonged exercise. In healthy marathon runners, hs-cTnT (median ∼31–46 ng/L, with >90% exceeding the 14 ng/L cut-off), CK (typically 1000–3000 U/L, occasionally up to ∼7000 U/L), and myoglobin (usually 500–2000 µg/L) frequently rise post-race and generally normalize within 72 h.^20–27^

Younger athletes often exhibit more pronounced but short-lived biomarker responses,^27^ suggesting that this rapid peak-and-decline pattern reflects transient metabolic changes rather than true cardiomyocyte necrosis.^25^

Supporting this, combined trials using biomarkers, echocardiography, and cardiac MRI have shown that troponin, CK, and myoglobin increase in all athletes after a marathon. Right ventricular systolic function may transiently decrease, and diastolic abnormalities can persist for up to 1 week, whereas CMR demonstrates no delayed myocardial enhancement—indicating that these biomarker elevations primarily reflect cytosolic release rather than true myocardial injury.^28^

Observational studies further indicate that cTnT elevations correlate with marathon completion time, while changes in creatinine, CK, and myoglobin show no clear relationship with age, sex, body mass index (BMI), training level, or prior marathon experience.^22^

Amateur runners display similar biomarker trends as elite athletes, though greater heterogeneity in lifestyle, cardiovascular risk, and training may increase susceptibility to adverse cardiac effects.^23^ Despite this, evidence for long-term harm in amateurs is insufficient. Baseline cardiac assessment, including a medical history, the cardiovascular risk profile, and a resting ECG, is recommended before intensive endurance training.^23^

Persistent or markedly elevated biomarkers exceeding typical post-race ranges and lasting 48–72 h warrant further evaluation. When combined with symptoms or abnormal imaging, they strongly suggest an underlying pathology, such as myocarditis.^2,20,24,26,29,30^

### Role of imaging and follow-up

Cardiac MRI, following the revised 2018 Lake Louise criteria, remains the gold standard for non-invasive diagnosis of myocarditis, risk stratification, and follow-up.^1,2,29,31^

Follow-up cardiac MRI should be performed within the first 6 months to assess residual inflammation or scarring as part of personalized management. The timing of MRI and other follow-up investigations should be individualized according to the patient’s symptoms, biomarker trends, ventricular function, and imaging findings.^2^

Follow-up cardiac MRI typically demonstrates marked improvement in myocardial oedema and a reduction in LGE within 6 months, whereas complete normalization during this time frame is distinctly uncommon. In a multicentre study of patients with myocarditis, ∼11% achieved full resolution of both oedema and LGE at 6 months, while most patients continued to exhibit LGE, which has been associated with a higher risk of adverse cardiac events and may indicate permanent myocardial fibrosis.^32^

Return to competitive sports should be individualized based on complete remission, defined by resolution of symptoms, normalization of biomarkers, and absence of active inflammation on imaging.^1,2,31^ A minimum restriction of physical activity for at least 1 month is recommended for both athletes and non-athletes, with the exact duration adjusted according to the patient’s clinical course.^2^ Follow-up after resuming activity should be individualized and may include repeated clinical evaluations, laboratory testing, ECG, ambulatory rhythm monitoring, and imaging studies. Annual follow-up is generally recommended in athletes due to the risk of recurrence or late sequelae but should be tailored to the patient’s clinical course and risk profile.^1,2^

### Use of non-steroidal anti-inflammatory drugs in amateur athletes

The use of NSAIDs, such as ibuprofen, shortly before the marathon may have aggravated an underlying myocardial inflammation in this patient. Although NSAIDs are commonly used by athletes to manage musculoskeletal complaints, experimental data indicate that they may impair myocardial healing and increase the risk of adverse cardiac events in the setting of acute or evolving myocarditis. In murine models of viral myocarditis, NSAID administration was associated with higher mortality, more extensive myocardial necrosis, and exacerbated myocardial inflammation, primarily via prostaglandin inhibition and modulation of immune responses during the early viremic phase of infection.^33^

Clinical observations in humans remain limited, but the 2025 ESC guidelines explicitly caution against NSAID use in suspected myocarditis due to concerns about delayed resolution of inflammation and increased arrhythmogenic potential.^2^

In this case, ibuprofen was taken the day prior to symptom onset for a musculoskeletal complaint, raising the possibility that it aggravated a subclinical myocarditic process following the recent respiratory infection. This temporal association, in the absence of other triggers, warrants consideration and underscores the need for caution with NSAID use in endurance athletes, particularly in the setting of recent infection or prodromal symptoms.

## Summary

This case illustrates that while transient biomarker elevations are common after marathon running, persistent or extreme elevations with imaging abnormalities indicate pathology. Adherence to guideline-based diagnostic and management strategies is essential for safe return to sport and optimal outcomes in young athletes with myocarditis.

In this patient, the clinical course was very favourable, with complete normalization on follow-up cardiac MRI after 3 months, highlighting that early diagnosis and appropriate management can lead to excellent recovery.

## Lead author biography


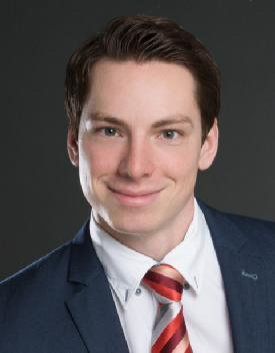


Jan Alphard Kleeberger, MD, MHBA, is an attending physician and group leader at the University Hospital Zurich and the Center for Translational and Experimental Cardiology (CTEC). A cardiologist and translational researcher, his work focuses on the role of non-coding Ribonucleic acid (RNAs) in heart failure, with particular emphasis on deep phenotyping. He received his clinical training in Germany and Switzerland at leading academic institutions. Dr Kleeberger is passionate about mentoring the next generation of physicians and is committed to reducing the global burden of cardiovascular disease through integrated research and clinical innovation.

**Consent.** The patient has provided written informed consent for publication of this case report in compliance with the COPE guidelines. The patient understands that every effort will be made to ensure anonymity, but complete anonymity cannot be guaranteed. The patient also understands and agrees that the information will be published in a journal with worldwide readership and online availability and that consent may be withdrawn at any time prior to publication, but once published, withdrawal of consent is no longer possible.

## Data Availability

The data underlying this article are available in the article and in its online Supplementary material.
